# Attenuating the Biologic Drive for Weight Regain Following Weight Loss: Must What Goes Down Always Go Back Up?

**DOI:** 10.3390/nu9050468

**Published:** 2017-05-06

**Authors:** Christopher L. Melby, Hunter L. Paris, Rebecca M. Foright, James Peth

**Affiliations:** 1 Department of Food Science and Human Nutrition, Nutrition and Metabolic Fitness Laboratory, Colorado State University, Fort Collins, CO 80523, USA; james.peth@colostate.edu; 2 Department of Kinesiology, Indiana University, Bloomington, IN 47405, USA; hlparis@indiana.edu; 3 Anchutz Medical Campus, Department of Medicine, University of Colorado-Denver, Denver, CO 80045, USA; Rebecca.Foright@ucdenver.edu

**Keywords:** weight regain, energy gap, energy intake, energy expenditure, diet composition, exercise

## Abstract

Metabolic adaptations occur with weight loss that result in increased hunger with discordant simultaneous reductions in energy requirements—producing the so-called *energy gap* in which more energy is desired than is required. The increased hunger is associated with elevation of the orexigenic hormone ghrelin and decrements in anorexigenic hormones. The lower total daily energy expenditure with diet-induced weight loss results from (1) a disproportionately greater decrease in circulating leptin and resting metabolic rate (RMR) than would be predicted based on the decline in body mass, (2) decreased thermic effect of food (TEF), and (3) increased energy efficiency at work intensities characteristic of activities of daily living. These metabolic adaptations can readily promote weight regain. While more experimental research is needed to identify effective strategies to narrow the *energy gap* and attenuate weight regain, some factors contributing to long-term weight loss maintenance have been identified. Less hunger and greater satiation have been associated with higher intakes of protein and dietary fiber, and lower glycemic load diets. High levels of physical activity are characteristic of most successful weight maintainers. A high energy flux state characterized by high daily energy expenditure and matching energy intake may attenuate the declines in RMR and TEF, and may also result in more accurate regulation of energy intake to match daily energy expenditure.

## 1. Introduction

Obesity, now classified as a disease [1], has become a global health problem with attendant increased risk of other chronic diseases. The etiology of obesity is complex, but fundamentally results from excess energy availability relative to energy expenditure, subsequently leading to energy storage, largely in the form of triacylglycerol molecules in adipocytes. Many of the untoward consequences of obesity result from infiltration of adipose tissue by macrophages, resulting in a local and systemic inflammatory state [2,3]. The excess energy availability can also lead to accumulation of lipid metabolites in ectopic depots such as liver, skeletal muscle, and pancreas, which contributes to inflammation and cellular dysfunction [4,5]. A reduction in the excess body fat stores can significantly reduce the inflammatory state and lower risk for associated chronic diseases [6]. Successful treatment must fundamentally involve a shift in energy balance such that total cellular energy expenditure exceeds availability. While this concept of energy balance may appear relatively simple, it has many layers of complexity that should be understood by scientists and practitioners in their attempts to provide the best available approaches to treating obesity. Despite the many lifestyle treatment approaches available, long-term maintenance of lost weight is often not successful [7]. Weight regain after weight loss is an all-too-common phenomenon. Thus, the current review will focus on two important questions: (1) What metabolic adaptations make long-term maintenance of weight loss so difficult? and (2) What lifestyle interventions can be used to counter these metabolic adaptations that promote weight regain, thus affording overweight and obese individuals greater opportunity to achieve relatively “permanent” (rather than transitory) improvements in body composition and health status?

## 2. Dynamic Energy Balance

The first law of thermodynamics dictates that energy ingested (Ein) and available for cellular metabolism in excess of energy expended (Eout) will result in increased energy stores, and that body energy stores will decrease if energy expenditure exceeds the amount of energy consumed and available for cellular metabolism. This first law has been inappropriately applied in nutrition counseling settings to inform patients/clients as to the magnitude of weight loss they will experience based solely on the degree to which they restrict their energy intake. Regrettably, this approach does not recognize that changing the intake/availability side of the energy balance equation will invariably result in metabolic and/or behavioral changes on the energy expenditure side. When dieting, the magnitude of the initial energy deficit is diminished over time as energy expenditure decreases due to adaptive thermogenesis (discussed later) and the loss of respiring body mass in the form of fat and some lean tissue as well [8,9].

The second law of thermodynamics dictates that the cellular reactions that generate ATP are less than perfectly efficient. That is, a portion of the energy released from the oxidation of macronutrients will be lost as heat rather than conserved in the phosphoanhydride bonds of ATP molecules. In human metabolism, more energy is lost as heat than is conserved and available for biologic work such as muscle contraction. Energy efficiency maximizes ATP production relative to heat loss or relative to mitochondrial oxygen consumption. In human studies, energy efficiency is often crudely determined based on changes in energy expenditure relative to changes in body mass or fat-free mass (FFM). However, FFM includes a variety of different tissues and organs with widely differing rates of energy expenditure, so attempting to quantify energy efficiency based on FFM rather than the components of FFM can provide less than complete information [10].

There is significant inter-individual variability in energy efficiency in response to well-controlled, experimentally-induced energy excesses and deficits [11,12,13], likely linked to genetic heritability and/or gene regulation (epigenetics). This may help explain why some individuals are more susceptible than others to weight gain/regain in the face of similar perturbations of energy balance [14,15,16,17], although the specific mechanisms are elusive. In regard to genetics, human obesity is considered to be primarily a polygenic trait, as monogenic forms of obesity are rare. Numerous candidate genes and gene loci have been identified that are associated with increased risk of obesity in large genome-wide association studies [18,19,20], although the risk alleles identified explain only a small portion of the variance in body mass index (BMI). Epigenetic inheritance is not the result of alterations in the base sequencing within genes, but rather is the modification of gene expression by such processes as DNA methylation and histone modification. Little research has focused on epigenetics and successful long-term weight loss, but in a recent pilot study, Huang et al. [21] identified differential epigenetic patterns between obese and non-obese individuals, but little difference in epigenetic markers between normal weight—never obese subjects and successful weight loss maintainers. Still, given the familial resemblance in BMI, there is the likelihood that both genetic and epigenetic factors play a role in determining not only risk for obesity, but also contribute to the significant between individual variability in weight loss and weight loss maintenance. While there is substantial interest in both genetic and epigenetic risk of obesity transmitted from one generation to the next, these topics are beyond the scope of this paper. The interested reader is referred to several recent reviews [22,23,24].

## 3. Why Is Weight Loss So Difficult to Maintain?

It is estimated that only about 20% of individuals who experience significant weight loss are able to maintain their lost weight [7]. Data from participants in the Biggest Loser Television program demonstrate the difficulty in maintaining lost weight over time. The average weight loss of the 14 participants during the 30-week intervention was 58 kg, but six years later, the contestants had regained an average of 70% of their lost weight (41 kg) [25]. The lack of successful weight loss maintenance for many dieting individuals could largely stem from behavioral issues—the inability of the individual to permanently adopt long-term lifestyle habits that promote a reduced body weight in the face of an obesogenic environment. The availability of a highly palatable, relatively inexpensive food supply coupled with a living environment that requires little physical work to carry out daily tasks could stand in the way of permanent changes in dietary and physical activity patterns. Lack of successful treatment then would stem from the inability to permanently alter behavioral responses to environmental conditioning and pressures. However, there is evidence that metabolic factors can contribute substantially to poor treatment outcomes—in response to weight loss, regulatory physiological responses are invoked that can effectively work to re-establish positive energy balance leading to weight regain toward a pre-established body weight set point [26]. While the voluntary behavior (conscious choices) versus biological determinism (pre-programmed set-point with tight control) debate is of keen interest to researchers in the field, the two are not mutually exclusive—behavioral and metabolic factors are inextricably intertwined, and both pose significant obstacles to long-term weight loss maintenance.

### 3.1. The Energy Gap Concept

**Figure 1:**
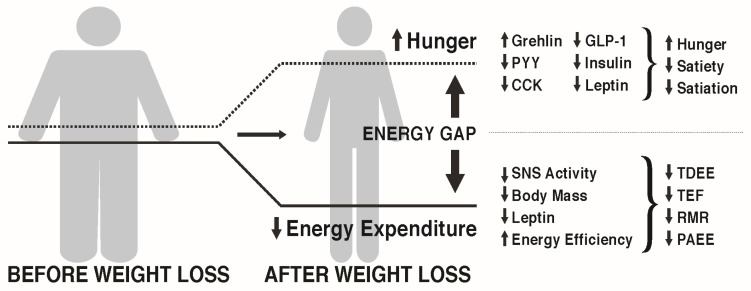
Energy intake and energy expenditure are balanced in weight stable obesity. In response to an energy-restricted diet, the resulting energy deficit and the weight loss together result in increased hunger and reduced energy expenditure. The discordance between energy desired and energy required is termed the energy gap. Altered circulating peripheral factors (increased orexigenic and decreased anorexigenic peptides) communicate a state of nutrient deprivation to the brain, resulting in increased hunger and increased food cravings, as well as a lower level of satiation. On the expenditure side, diet-induced weight loss causes a significant reduction in total daily energy expenditure (TDEE) owing to reduced resting metabolic rate (RMR) and the thermic effect of food (TEF), and often decreases in exercise energy expenditure and non-exercise activity thermogenesis. The increased hunger and decreased energy expenditure can readily promote weight regain, especially in the face of an obesogenic environment characterized by an abundance of high energy dense food and little need to engage in physical activity. PYY: peptide YY; CCK: cholecystokinin; GLP-1: glucagon-like peptide-1; PAEE: physical activity energy expenditure.

MacLean et al. [27,28] and others [29] have reviewed the metabolic adaptations that accompany weight loss, primarily based on data from animal studies, but with probable relevance to the human condition. As depicted in Figure 1, calorie restriction leading to weight loss causes discordance between appetite and energy requirements, a concept referred to as the “energy gap“, in which the biologic pressure to regain the lost weight occurs as a function of the increased hunger and the reduced energy expenditure that accompany diet-induced weight loss. In response to weight loss, signals of both energy and nutrient deprivation are sent from the periphery to brain networks in the hypothalamus and hindbrain, which by way of second order neurons increase hunger and decrease energy expenditure, resulting in more calories being desired (Ein) than are required (Eout). The occurrence of these responses among individuals living in an obesogenic environment can promote the re-establishment of positive energy balance and regain of body weight and fat toward their pre-diet levels.

### 3.2. How Does Weight Loss Affect Hunger and Satiety?

Many metabolic factors contribute to the energy gap following dietary restriction. Observed changes in adiposity-related signals (leptin and insulin), hypothalamic neuronal activity and neuropeptide expression, and gut peptide expression are thought to play a role in the increased hunger in response to weight loss. An exhaustive review of these factors is beyond the scope of this paper. The interested reader is referred to comprehensive reviews on this topic [14,27,28,30].

The hypothalamus integrates many signals from the periphery, including liver, gut, and adipose tissue to regulate energy expenditure and the initiation, termination, and frequency of eating. Homeostatic regulation of food intake occurs such that severe caloric energy restriction leading to weight loss results in a strong internal drive to eat, whereas an overabundance of food intake and weight gain may be followed by a reduction in food intake. Insulin and leptin are putative players in this regard [31,32,33,34]. They circulate in proportion to body fat mass and bind receptors in hypothalamic neurons, promoting expression of the anorexigenic peptides, pro-opiomelanocortin (POMC) and cocaine-amphetamine related transcript (CART), and inhibiting expression of the orexigens, neuropeptide Y (NPY) and Agouti-related peptide (AgRP). Acting through second-order neurons, these leptin- and insulin-stimulated neuropeptide changes result in reduced food intake and increased sympathetic nervous system activity and energy expenditure [31,34]. Because increasing fat mass results in incremental increases in circulating leptin, the majority of obese children and adults present with hyperleptinemia [35]. However, this elevation in blood leptin concentrations occurs without an ensuing decrease in food intake, indicating the presence of leptin resistance among individuals who exhibit obesity. While the higher circulating leptin and insulin coincident with increasing adiposity should theoretically limit weight gain, cellular resistance to these hormones occurs suggesting that protection against weight gain may be less robust than protection against weight loss [32]).

Speakman [36,37,38] has argued that human survival against risk of starvation (i.e., body energy stores insufficient for reproduction and life) and risk of predation (i.e., excess body mass impairs the ability to escape predators) is delimited by dual (upper and lower) intervention points and environmental and behavioral pressures exert the primary influences on body weight within the range between them. In accord with this view, for most individuals who exhibit body weight between the upper and lower intervention points, the availability of food would be a primary determinant of food intake. However, as weight falls outside this range, genetically-driven (and/or epigenetic) physiologic changes occur that promote restoration of body energy stores that support survival. Therefore, when inadequate food availability (whether due to intentional energy restriction, as with dieting, or involuntary severe energy deficit, as with famine) results in weight loss that reaches the lower intervention point, homeostatic metabolic changes are invoked that promote weight regain. Conversely, as body weight increases (in today’s society, this would be largely due to the obesogenic environment) and reaches the upper intervention point, homeostatic adjustments should theoretically come into play to decrease energy intake and increase energy expenditure, thus limiting weight gain and risk of predation. However, Speakman [36,37,38] argues that owing to a substantial reduction in predatory risk within the human population today, genetic shifts have occurred such that there is a decidedly less robust defense against weight gain than weight loss. Weight gain that exceeds the upper intervention point produces, at best, only modest reductions in hunger and increases in energy expenditure, in part due to the aforementioned leptin resistance. On the other hand, when body weight falls below the lower intervention point, leptin and insulin rapidly decrease causing increased food intake and reduced energy expenditure. This is not unexpected given the decrease in fat mass. However, the magnitude of the decrement in circulating leptin is much greater than the magnitude of fat loss [39], a phenomenon that may be one of the primary drivers of weight regain. During weight loss maintenance, leptin concentrations slightly increase relative to the dynamic weight loss state [35,40]; however, these levels remain significantly reduced even when adjusted for changes in fat mass after one [39,40] and two years of weight maintenance [41].

A host of other anorexigenic peptides originate in the gut and typically increase in circulation in response to feeding, which then communicate with the hypothalamus to terminate food intake, increase satiety, and increase satiation between meals [42,43,44]. These peptides include cholecystokinin, peptide YY, amylin, pancreatic polypeptide, and glucagon-like peptide-1 (GLP-1). There is increasing evidence of a sustained, long-term decrease in anorexigenic signals in response to diet-induced weight loss, with the decrement being greater than the decline in body weight [40]. Such physiological changes could result in a metabolic milieu that readily promotes weight regain following weight loss.

Ghrelin is an orexigenic hormone, primarily produced by oxyntic cells of the stomach and is the endogenous ligand for the growth hormone secretagogues receptor type 1a (GHS-R1a) [45,46]. The GHS-R1a is located throughout the body including the hypothalamus, pituitary, neuroendocrine tissues, pancreas, stomach, and vagus nerve [47]. The presence of intact vagal afferents is essential for the centrally mediated effects of ghrelin on hunger and satiety. In lean individuals, plasma ghrelin concentrations rise during fasting and drop with meal ingestion proportional to the calorie content of the meals [48]. Obese individuals may not display the same suppression of ghrelin in response to calorie ingestion [49,50]. Weight loss leads to an elevation of plasma ghrelin in obese adolescents [51] and adults [52], which is thought to be a compensatory adjustment designed to increase energy intake in an attempt to return body fat stores to their initial levels. The available data suggest that the increase in circulating ghrelin that accompanies weight loss and persists into the weight maintenance phase could contribute to increased hunger and the energy gap.

Additionally, compared with normal-weight individuals with no history of obesity, individuals who are overweight or obese, but have lost weight, have a different neural response to overfeeding [53]. In a randomized crossover study involving a two-day eucaloric feeding condition and a two-day 30% overfeeding condition, Cornier and colleagues [53] used Functional Magnetic Resonance Imaging (fMRI) to compare the neuronal responses to viewing images of food among “thin” participants—normal-weight individuals (BMI 19–23 kg/m^2^) with no history of obesity—and “reduced-obese” participants, who were overweight or obese (BMI 27–32 kg/m^2^) but recently lost weight in a weight-loss program. The study was designed to analyze brain responses to food images in the overfed state versus eucaloric state. Among thin individuals, overfeeding attenuated neural activation compared to that observed during the eucaloric state. This response to overfeeding did not occur among reduced-weight overweight/obese individuals. In the baseline fasting state, thin individuals had a much more robust neuronal response to food-related visual cues than reduced-obese individuals. Overfeeding resulted in significant attenuation of the response to visual foods cues in thin but not reduced-obese individuals.

Much of the research on body weight/composition regulation has been adipocentric—that is the control of energy intake and expenditure has focused on adiposity-related signals as discussed above. However, recent studies have demonstrated that fat-free mass (FFM) and resting metabolic rate (RMR) are positively associated with energy intake [54,55]. FFM is the strongest predictor of RMR and this relation suggests the possibility of a link between FFM-driven energy requirements and the homeostatic control of energy intake. In other words, the large amount of metabolically active lean tissue found in most obese individuals could provide signals to drive the high energy intake necessary to sustain the obese state. However, there is also evidence that the FFM depletion (along with loss of body fat) resulting from caloric restriction fails to dampen appetite, but instead contributes to hyperphagia. Dulloo et al. [56,57] have suggested that the relation between FFM and energy intake is, in fact, U-shaped, such that the large FFM associated with obesity ‘passively’ drives high energy intake, but the decrement in FFM associated with diet-induced weight loss enhances the drive to eat. Further, they suggest that the reduced FFM that occurs with weight loss stimulates increased energy intake in order to restore the FFM, but also causes increased fat deposition, a phenomenon they describe as ‘collateral fattening’. If during weight regain following weight loss, the restoration of FFM lags behind fat restoration, hyperphagia could persist beyond the fat mass “catch up” and result in greater body fat storage than existed prior to dieting. Thus, weight loss-induced reductions in FFM could contribute to both aspects of the energy gap—a reduction in energy expenditure and increased hunger, both of which could contribute to weight regain.

Taken together, diet-induced weight loss appears to impact appetite via changes to both fat mass and FFM by altering known and unknown peripheral factors that communicate to the brain a state of nutrient deprivation. These changes result in increased hunger [58], which is further exacerbated by increased food cravings in response to diet-induced weight loss [59], especially from foods of higher energy density [59], and a lower level of satiation in response to overfeeding when compared to never obese individuals [53]. The above discussion is not to say that body weight and body composition are necessarily tightly controlled in all humans and that all weight regain is entirely a biologically-driven process. Indeed, the rapid population increase in the prevalence of obesity associated with the increasingly obesogenic environment would argue against exquisitely tight control, especially in regard to protection against weight gain.

### 3.3. How Does Weight Loss Affect Energy Expenditure?

Total daily energy expenditure (TDEE) is a function of four components: resting metabolic rate (RMR: the energy expenditure required for cellular processes necessary for life as measured when an individual is lying quietly and awake in a post-absorptive state), the thermic effect of food (TEF: the increase in energy expenditure above RMR in response to food ingestion), non-exercise activity thermogenesis (NEAT: energy expenditure above RMR required to support the activities of daily living as well as fidgeting), and exercise energy expenditure (ExEE: energy expended above RMR necessary for performing exercise). NEAT and ExEE together make up physical activity energy expenditure (PAEE). Diet-induced weight loss almost always causes significant decreases in TDEE, which can negatively impact the maintenance of lost weight. As far back as 1984, Leibel and Hirsch reported lower TDEE in post-obese compared to never-obese individuals—on the order of 25% lower than predicted by metabolic body size [60]. In a later study, they determined that obese subjects undergoing 10–20% weight loss experienced a significant decrement in TDEE which could not be entirely explained by the loss of respiring body mass [61]. They reported a mean reduction in TDEE of 8 kcal/kg fat-free mass per day in those obese subjects who lost at least 10% body weight. These decreases in TDEE reflect adaptive thermogenesis (AT)—the change in energy expenditure independent of changes in FFM and the composition of FFM. AT may persist long term [25,61].

The TEF is reduced with dieting because there is a reduction in the total caloric load that requires obligatory digestion and absorption. A portion of the normal TEF results from increased sympathetic nervous system activity that accompanies food ingestion [62], which also decreases with weight loss and lower quantities of food intake.

Numerous studies have shown that weight loss-induced declines in RMR contribute to the reduced TDEE. The reduction in RMR is partially the result of the loss of respiring body mass, but many studies [25,63,64,65], but not all [66], report the magnitude of the decline to be greater than can be explained by the reduction in respiring mass. In the case of diet-induced negative energy balance, a high AT characterized by a reduction in energy expenditure disproportionate to the reduction in FFM (increased resting energy efficiency) attenuates the weight loss drive. The AT associated decrement in RMR could be due to changes in FFM composition, decreases in sympathetic nervous system activity, and lower circulating tri-iodothyronine, leptin, and insulin. Note, however, the magnitude of AT and its contributors appear to vary significantly between individuals and also according to the phase of weight loss. Muller et al. [10] point to the importance of characterizing differences in adaptive thermogenesis during on-going (active) weight loss versus fixed weight loss (maintenance). They suggest that the reduced insulin concentration and changes in the composition of FFM (reduced glycogen and intracellular water) are the primary drivers of AT in the RMR during the first several days of active weight loss (phase 1), but in the second phase as the velocity of ongoing weight loss slows there is little to no AT and the continuing decline in RMR is largely a function of decreased FFM.

There is substantial evidence of AT in regard to RMR with weight loss. For example, Leibel et al. found that a 10–20% weight loss in obese patients caused a decrease in RMR of 3–4 Kcal/kg fat-free mass per day [67]. In the study of Biggest Losers participants, despite the mean weight regain of 41 kg during the six years following the competition, mean RMR of the participants remained 700 kcal/day lower compared to baseline and no different compared to the end of the intervention at 30 weeks [25]. Notwithstanding the sizable inter-individual variation in the magnitude of RMR responses and use of different metabolic carts to measure RMR at six years compared to baseline and 30 weeks, these data provide evidence of significant long-term metabolic adaptation (increased metabolic efficiency) that accompanies weight loss. Even among athletes undergoing significant exercise training, weight loss may result in a decrement in RMR that is disproportionate to the loss of body mass [63,68]. Note that during weight loss maintenance (fixed weight loss) AT is present, with TDEE lower than predicted based on metabolic body size, with this decrement due primarily to the non-resting component as explained below.

The effect of diet-induced weight loss on ExEE and NEAT is quite variable. From a thermodynamic perspective, without changes in movement efficiency and economy, the loss of body mass will result in fewer calories expended to perform the same weight bearing movements. When considering the energy expenditure incurred from activities of daily living at the lower body weight, this could conceivably contribute substantially to the reduction in total daily energy expenditure. Indeed, following a 10% weight loss, individuals were found to exhibit greater skeletal muscle work efficiency at lower intensities, which Rosenbaum et al. estimated could account for one-third of the reduction in PAEE [69]. Others have also found weight loss to result in increased energy efficiency at low exercise workloads [70,71], possibly resulting from the decrease in circulating leptin [70]. There is substantial inter-individual variability regarding changes in energy expenditure and efficiency (i.e., adaptive thermogenesis) with weight loss, both of which are associated with the aforementioned reductions in circulating insulin and leptin. However, two recent studies suggest that changes in the magnitude of circulating leptin and insulin sensitivity alone are not sufficient to explain the magnitude of weight regain [72,73].

To summarize the changes in energy expenditure, diet-induced weight loss can result in significant reductions in RMR, TEF, ExEE, and NEAT. These metabolic changes that result in lower energy expenditure would not obligatorily contribute to weight regain if there were a proportional decrease in food intake. However, as discussed earlier, body composition and hormonal changes occur with weight loss that are associated with increased rather than decreased appetite. This scenario coupled with the environmental pressures of modern society that favor excessive calorie consumption and minimize physical activity, could lead to a sense of futility among both patients and practitioners regarding lifestyle obesity treatment. However, as MacLean et al. [27] suggest, the energy gap “should not be misconstrued into a conciliatory surrender to the inevitability of weight regain”. They instead indicate that the “biological drive to regain lost weight can be countered with environmental, behavioral, and pharmaceutical interventions”. However, they stop short of providing such information in their review.

Thus, the purpose of this second section is to examine lifestyle approaches to attenuate the increase in hunger and the decrease in energy expenditure that accompany weight loss and to identify important areas for further research.

## 4. Can the Weight Loss-Induced Energy Gap Be Attenuated by Lifestyle Factors to Enhance Weight Maintenance?

**Figure 2:**
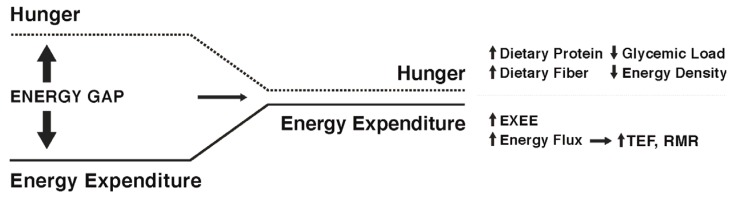
To increase the probability of long-term weight loss maintenance, narrowing the energy gap by reducing hunger and increasing energy expenditure following weight loss is of critical importance. Increased dietary protein and fiber, and reduced energy density and glycemic load may be helpful in reducing hunger and enhancing satiation between meals. Increased exercise energy expenditure can contribute to increased total daily energy expenditure as long as compensatory reductions in NEAT do not occur. The higher TDEE allows greater energy intake without weight regain, a state called high energy flux. The latter has been shown to contribute to increased TDEE by increasing resting metabolic rate (RMR), and the higher energy intake results in a higher thermic effect of food (TEF).

While some individuals are genetically more susceptible to obesity and will likely find it difficult to maintain weight loss [74], there is evidence to suggest that specific strategies and approaches can increase the probability of success. Studies of participants enrolled in the National Weight Control Registry (NWCR, in which enrollees have successfully maintained at least a 13.6 kg weight loss for a minimum of one year, with an average of 33 kg loss maintained for five years [7]) have been useful in identifying predictors of successful weight loss maintenance versus weight regain [75,76,77,78,79,80]. Weight regain has been associated with higher levels of depression, binge eating, dietary disinhibition, increases in hunger, and higher percentage of energy ingested from fat. Predictors of successful weight loss maintenance in the NWCR cohort include frequent weight monitoring, high levels of physical activity, reduced time spent in sedentary activities including television viewing, high levels of dietary restraint, and lower calorie and fat intake. In the most recent longitudinal follow-up of NWCR participants [81], 87% maintained at least a 10% weight loss over a 10-year period. The greatest weight regains occurred in participants who reported large decreases in dietary restraint, physical activity levels, and self-weighing frequency during the first year after weight loss. Note, those enrolled in the NWCR are not representative of all individuals who have attempted weight loss and who have experienced long-term maintenance, but rather are self-selected based on their success. Nevertheless, some findings from the NWCR are supported by those from the MedWeight registry in Greece, which includes both weight loss maintainers and regainers [82,83,84,85,86]. Among male registrants, weight loss maintainers were found to exhibit greater physical activity (approximately 200 kcal/day more than regainers), greater adherence to a dietary pattern emphasizing home-cooked meals, fruits, vegetables, unprocessed grains, nuts, lean proteins, low-fat dairy products, and olive oil, and lower daily intakes of salty snacks, sugary soda beverages, and alcohol. Among the women, weight maintainers ate more slowly and slightly more often, and consumed greater protein intakes relative to body mass. In another recent study [87], women who had lost a minimum of 10% body weight loss for at least one year reported higher protein and lower carbohydrate intakes and more vigorous physical activity compared to women who had lost and regained at least 10% body weight. In all of these studies, maintainers versus regainers could differ in a variety of genetic, epigenetic, psychological, and other factors. Nevertheless, these data provide evidence that maintenance of lost weight can and does occur in some individuals, even in the face of environmental pressures and well-recognized metabolic adaptations. Figure 2 depicts possible ways to narrow the energy gap by decreasing hunger and increasing energy expenditure.

## 5. Approaches to Attenuate the Increased Hunger Following Weight Loss

There currently are no clear-cut dietary recommendations for successful weight maintenance following weight loss. In experimental studies examining dietary approaches to curb appetite during weight maintenance, there is substantial inter-individual variability in response to the different diets, suggesting that a one-size-fits-all weight maintenance dietary approach is not likely to be effective for all. Clearly, there is need for more experimental research on dietary patterns and weight maintenance, with special focus on approaches tailored to the unique behavioral and metabolic characteristics of the individual. While much research has focused on understanding the biologic basis for changes in hunger and satiety associated with acute energy perturbations, fewer studies have addressed experimental approaches to reduce hunger over the long-term following weight loss. With this caveat in mind, the following sections provide a brief review of the possible role of diet composition in attenuating the increased hunger that occurs with weight loss. There are other dietary features that might also be useful in minimizing weight regain such as time restricted feeding to better match meal consumption with circadian rhythms [88,89], altering the gut microbiome [89], and consuming water or soups prior to meals [90,91,92], but a discussion of these is beyond the scope of this review.

### Diet Composition

Diets of varying macronutrient composition (e.g., high protein; high carbohydrate, low fat; low carbohydrate, ketogenic diets) for weight loss have been the focus of significant research in the past two decades. However, the possible effects of dietary macronutrient composition on weight maintenance following weight loss have received far less research attention, especially in regard to long-term (years) results. Theoretically, a dietary pattern that enhances weight loss maintenance by attenuating the energy gap would result in less hunger and greater satiation relative to energy intake and less reduction in energy expenditure relative to energy intake. Given the greater satiating value of protein compared to other macronutrients [93,94,95] along with its higher thermic effect upon consumption [62], higher intakes of protein may be especially helpful for weight maintenance. Several studies provide experimental evidence in support of the theoretical effects. Westerterp-Plantenga et al. [96] reported that following 5–10% weight loss in overweight and obese men and women, the addition of 48 g/day of protein to the usual dietary intake (total of 18% of energy intake as protein) compared to no additional protein added to usual intake (15% of energy intake as protein) during a three-month weight maintenance period resulted in only half the body weight regain, with the weight regain consisting of fat-free mass. The ‘additional protein’ group also experienced increased satiety and decreased energy efficiency, both of which are important in attenuating the energy gap. In a similar study by the same research group [97], following a 7.5% weight loss in overweight individuals, the addition of 30 g/day of protein to the usual diet compared to no additional protein during a 6-month weight maintenance follow-up, resulted in significantly less weight regain (0.8 vs. 3.0 kg), improved body composition, and greater satiety. In a recent review focused on dietary protein and body weight regulation, the authors suggest daily protein intakes between 1.2 g·kg^−1^ and 1.6 g·kg^−1^, with each meal providing at least 25–30 g protein. The greater satiety and better weight maintenance outcomes seen with higher protein intakes [98] may be related to protein-induced secretion of anorexigenic hormones including GLP-1 and PYY and suppression of the orexigenic hormone ghrelin [99,100,101].

Another aspect of dietary macronutrient composition that may have relevance to attenuation of the weight loss-induced energy gap is the glycemic load, a function of the glycemic index (mathematically derived numerical expression of the ability of the carbohydrates in food to raise blood glucose concentrations following ingestion) multiplied by the total amount of carbohydrate in the diet. Observational studies described in a prior section have found weight loss maintenance to often be associated with consumption of low energy dense foods including vegetables, whole fruits, and legumes, and low carbohydrate foods such as lean meats, which together would constitute a low-to-moderate glycemic load eating pattern. Experimental studies have shown that meals with lower energy density result in lower energy intake [102]. However, experimental studies examining low glycemic load diets and weight maintenance are few, with the exception of low-carbohydrate diets, which are by definition low glycemic load diets. These have been proven in experimental studies to be effective for weight loss, often more so than low fat diets [103,104] in head-to-head comparisons over 3–6 months. Individuals following low-carbohydrate weight loss diets report being less bothered by hunger than those on low fat diets [105], but still long-term dietary adherence to extremely low-carbohydrate diets does not appear much better than other dietary approaches [103], so their utility for weight maintenance is questionable. Notwithstanding issues of dietary adherence, using a randomized three-way cross-over design, Ebbeling et al. [106] found that following 10–15% weight loss in overweight and obese adults, the declines in RMR and TDEE were attenuated most by isocaloric low-carbohydrate and low-glycemic load diets, respectively, compared to the low-fat dietary condition when consumed during 4 weeks of weight loss maintenance.

A number of epidemiological studies have found high fiber intakes to be associated with lower food intake and improved weight control [107,108,109]. High fiber diets are thought to evoke a time-energy displacement, which refers to the increased time it takes to chew and consume indigestible carbohydrates (fiber). Slowed eating may allow more time for gastrointestinal peptides to be released during the meal, resulting in earlier meal termination. Related to this concept, meals high in fiber exhibit lower energy density which may decrease energy intake over the course of the meal. Duncan et al. [110] demonstrated that when participants ate a low energy density diet over five days as compared to a five-day high energy density diet they spent 33% more time eating yet achieved satiety at approximately half the energy intake. Some high fiber meals also increase gastric distention and slow gastric emptying [111] which have been associated with increased satiety [112], may increase postprandial concentrations of anorexigenic gastrointestinal peptides including GLP-1 and PYY [113], and may alter the gut microbiome by increasing the abundance of bacteria capable of fermenting dietary fibers to produce short-chain fatty acids that are associated with increased satiation by stimulating greater secretion of GLP-1 and PYY. These effects of dietary fiber could help explain the association between lower energy density dietary patterns and successful weight loss maintenance in the NWCR participants. However, many clinical studies have failed to provide support for a role of fiber in reducing food intake. In a recent systematic review of acute interventions, the majority of studies did not find increased satiety and decreased food intake with increasing fiber intake [114]. The disparity between the epidemiological findings and many clinical trials might result from differences in the types and amounts of fiber given, the short treatment periods (≤24 h), or the inherent difficulties in translating the results of a laboratory test of hunger, satiety, and ad libitum food intake to a real-world setting.

## 6. Approaches to Attenuate the Decline in Energy Expenditure Following Weight Loss

While exercise is the most obvious volitional approach to increasing energy expenditure, the different constituents of TDEE each represent a potential target for interventions.

### 6.1. Resting Metabolic Rate

Because RMR accounts for upwards of 75% of TDEE, attenuating its decline could have major implications for weight maintenance. Elevating total caloric turnover while maintaining energy balance may be one possible method for attenuating the fall in RMR. We have previously seen in young males [115], females [116], and older adults [117] that high energy flux (high levels of energy expenditure, achieved by significant daily exercise training, matched with high daily energy intake such that energy balance is maintained) is associated with higher RMR values. We have also shown that RMR decreases significantly when energy balance is maintained with cessation of exercise and simultaneous reduction in energy intake [118]. Thus, the higher RMR seen with high exercise energy expenditure and intake appears to be a transient phenomenon, indicating the importance of regular, chronic exercise. Because fat-free mass is the major contributor to resting metabolism, increasing skeletal muscle through resistance training represents another approach to elevating RMR. However, the contribution of skeletal muscle to RMR is far less than that of the internal organs [119,120]. Furthermore, with the exception of trained athletes, most individuals who engage in resistance exercise produce insufficient gains in skeletal muscle mass to exhibit appreciable increases in RMR [121]. However, resistance exercise could contribute to a high energy flux state and we have shown that both men [122] and women [123] undergoing an acute strenuous resistance exercise bout exhibit significantly higher RMR values measured 15 h later. Of interest is that among NWCR members, 24% of men and 20% of women reportedly engage in weight lifting on a regular basis [124].

### 6.2. Thermic Effect of Food

Macronutrients differ in the thermic effect they elicit, with fat having the lowest thermic effect at approximately 3% of ingested fat calories, carbohydrates producing a thermic effect of 5–10% of ingested carbohydrate calories, and protein consumption eliciting a thermic response of 20–30% of protein calories [62]. Therefore, while reducing caloric load will inevitably lower TEF, food selection could offset some of this reduction, with meals higher in protein potentially resulting in a greater thermic response [125]. Not only can meal composition influence TEF, but meal duration may also alter the thermic response, with slowed meal consumption having a greater TEF compared with rapid meal consumption [126]. Still, given the relatively small contribution of TEF to TDEE, higher protein consumption could modestly elevate TEF without significantly raising TDEE [127,128].

### 6.3. Non-Exercise Activity Thermogenesis

Total daily energy expenditure varies widely, even among individuals of similar age and body size [129] and because RMR is largely determined by body size, and TEF contributes minimally to TDEE, the variance among individuals of similar body size is largely attributed to differences in physical activity thermogenesis, which includes both NEAT and ExEE. As previously described, diet-induced reductions in body mass will cause increased metabolic efficiency at low work intensities characteristic of NEAT [69,70,71]. Added to this increased energy efficiency are the possibilities that the amount of activity may be reduced with weight loss, and initiating a program of exercise to enhance energy expenditure could result in compensatory decreases in NEAT [130]. On the other hand, whereas a larger body mass prior to weight loss may restrict some activities, the post weight loss condition may afford less burdensome movement and greater activity at work and during leisure time. Levine et al. [129] have described this in a previous review in which they show a hypothetical example of how a transition from a sedentary to an active lifestyle might result in an elevation of NEAT by over 1000 kcal per day. While this scenario may not apply directly to a hypometabolic state following weight loss, the notion that alterations in NEAT may have wide-ranging effects remains the same—namely, that reductions in NEAT due to weight loss-induced changes in movement efficiency and economy may be counterbalanced by the elevations in NEAT resulting from increased activity such as more walking, taking stairs, spending less time sitting, etc.

### 6.4. Exercise Energy Expenditure

Exercise typically produces less energy deficit and less weight loss compared to energy-restricted diets, and its impact on body weight regulation has been questioned [131]. Nevertheless, participation in regular exercise is one of the strongest predictors of weight loss maintenance [132,133]. Energy expenditure may be elevated through a variety of exercise approaches, and while prescribing specific exercise strategies for weight loss maintenance is beyond the scope of this review, approaches that are time efficient and motivating may be more sustainable for participants. High-intensity interval training (HIIT) may be one such approach for some individuals. HIIT alternates short bursts of intense exercise with recovery periods and may serve as an effective time-efficient replacement to endurance-based training. For example, Sevits et al. [134] found that a single exercise session consisting of five 30-second bouts of maximal effort cycling exercise, separated by 4 min resting intervals (2.5 min of actual exercise during ~20-min period) produced an average net energy cost of 225 kcal—an increase in TDEE by approximately 10%. Performing longer duration intervals (~4 min) at 80–95% VO2max for 3–6 months improves BMI and body fat percentage in overweight/obese individuals [135], and may be more effective at improving cardiometabolic risk factors than moderate intensity training [136]. Less is known regarding the impact of HIIT on weight loss maintenance, but through its alteration of the hormonal milieu and impact on fat oxidation, HIIT may contribute to an elevated energy expenditure that promotes sustained weight loss [137]. Apart from the greater energy expenditure resulting from an active lifestyle, regular exercise may contribute to weight maintenance by reduced efficiency of nutrient storage. Animal models have shown that weight regain results in the preferential storage of dietary fat and the utilization of calories derived from carbohydrate [138]. However, regular exercise enhances fat oxidation and glycogen storage, and when weight maintenance was accompanied with regular exercise, the shift in fuel utilization brought on by exercise altered the traditional method of energy restoration (storage of fats and utilization of carbohydrates) to a more energetically costly method [139].

## 7. Narrowing the Energy Gap by Increased Energy Flux: Effects on Both Appetite and Energy Expenditure

In 1956, Mayer and colleagues [140] observed a group of Bengali workers, and found that energy intake was more appropriately matched with energy expenditure in those workers whose occupation required greater amounts of physical activity. The workers with the highest occupational physical activity demands consumed more food energy, while those whose occupations demanded less movement, ate less. However, this was true only up to a point. Among those with sedentary occupations and lifestyle the low level of physical activity was not coupled to low food consumption, but on the contrary, was coupled to higher caloric intake [140]. Whereas an active lifestyle was associated with accurately regulated energy intake to match expenditure, a sedentary lifestyle was associated with discordance of the two and a widening of the energy gap. This observation has since been supported by data showing that a reduction in physical activity characteristic of a sedentary lifestyle fails to result in a compensatory reduction in energy intake [141].

A physically active lifestyle may be important not only for its contribution to energy expenditure, but also for its role in matching the energy desired with the energy the individual requires. Regular exercise may heighten the brain’s sensitivity to leptin and insulin, and enhance satiety signals coming from the periphery [142]. A substantial body of research literature exists suggesting that high intensity acute exercise results in a short-term suppression of appetite [143], often without a compensatory increase in calories for a day or two, possibly mediated by decreases in ghrelin and increases in anorexigenic peptides including PYY and GLP-1 [144]. However, the effects of chronic exercise on long-term appetite regulation are less well understood. Based on large variability of body weight and composition changes in response to a monitored exercise program lasting many months [145], the impact of long-term exercise on appetite and food intake is difficult to predict on an individual basis [146]. Clearly some individuals compensate more than others for the energy cost of exercise by consuming more calories [147], but the reasons are unclear, and the effect of exercise on appetite following rather than during weight loss is understudied.

**Figure 3:**
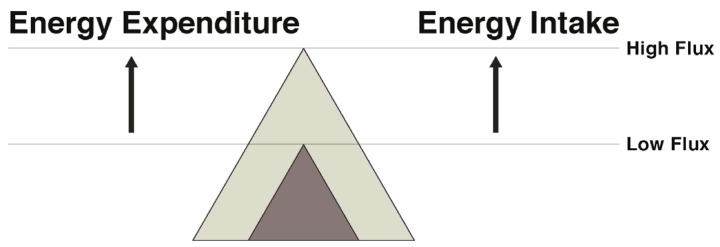
Following diet-induced weight loss, the reduced body weight could be maintained in a low energy flux or high energy flux state. The low flux state is characterized by low energy expenditure with matching low energy intake. However, with the increased appetite resulting from weight loss, this becomes difficult, especially in an obesogenic environment with abundant food availability. The high energy flux state is characterized by higher expenditure and higher energy intake, which is associated with less hunger and attenuation of the energy gap.

In regard to exercise and physical activity, the transition to energy balance during weight maintenance can be approached in several ways. Weight can be maintained by pairing a low energy intake with a low energy expenditure (low flux state), resulting in energy balance with a low total throughput of energy. Alternatively, energy balance can be maintained with a significantly greater energy throughput by coupling a higher expenditure via exercise with a higher intake (high energy flux) (Figure 3). While both result in energy balance, in the face of an array of palatable food available in our current society, remaining in energy balance in a low flux, sedentary state seems improbable for most. Rather, maintaining a high flux state appears to be a more promising way to attenuate the weight loss-induced energy gap.

One could question whether previously obese and/or sedentary individuals can achieve adequately high levels of ExEE to maintain a state of high energy flux. To answer that question, we recently conducted a study [148] in which obese individuals underwent 7% weight loss over several months followed by 3 weeks of weight maintenance at their reduced body weight. They were then asked to complete two separate flux states in random order for five days each: high energy flux (net ExEE of 500 kcal/day at 65% VO_2_ max with daily energy intake set at RMR (kcal) × 1.7 to maintain energy balance) and 5 days of low flux (no ExEE and limited physical activity with energy intake set at RMR × 1.35 to match the low TDEE). Mean RMR was significantly higher during high flux compared to low flux, suggesting that higher energy flux may help attenuate the decline in RMR in in obese individuals attempting to maintain lost weight. Also, resting fat oxidation was higher in the high compared to the low flux condition, which may have implications for fat balance. Importantly, while in the high flux condition, study participants reported significantly lower levels of hunger and greater fullness in comparison to their days spent in low flux, despite being in energy balance across the two different flux conditions [148]. Given the short period of time spent in the high and low flux states in our study, future research is needed to address this issue in a larger sample over a longer time period. Still, our findings are concordant with results from the Look Ahead Trial in which greater physical activity that was accompanied by higher food intake was associated with weight loss maintenance over a four-year period [149]. Also in support of the flux concept, a recent prospective study showed low energy flux significantly predicted body fat gains over several years in both adolescents and college-aged women [150].

## 8. Summary and Conclusions

Successfully maintaining lost weight is difficult for many obese and overweight individuals who diet to lose weight. Metabolic adaptations occur with weight loss that result in increased hunger, which is discordant with simultaneous reductions in energy requirements—producing the so-called energy gap in which more energy is desired than is required. These metabolic adaptations have been characterized, with the increased hunger and decreased satiety and satiation associated with elevation of the orexigenic hormone ghrelin and decrements in anorexigenic hormones including CCK, PYY, and GLP-1. The lower total daily energy expenditure that accompanies weight loss results from (1) a disproportionately greater decrease in circulating leptin and RMR than would be predicted based on the decline in body mass, (2) lower decreased thermogenesis owing to reduced food consumption, and (3) increased energy efficiency at work intensities characteristic of activities of daily living. These metabolic adaptations can readily promote weight gain, especially in the face of an obesogenic environment characterized by abundant access to highly palatable, energy dense food and low levels of physical activity energy expenditure. However, weight regain following weight loss should not be viewed as inevitable. While more experimental research is needed to identify effective strategies to narrow the energy gap and attenuate weight regain, factors contributing to long-term weight loss maintenance have been identified from observational studies and some experimental studies. Vigilant self-monitoring of body weight and reduced time in sedentary activities including television viewing appear especially important. Lower hunger and greater satiation have been associated with higher intakes of protein and dietary fiber, and lower glycemic load diets. High levels of PAEE are characteristic of the vast majority of successful weight maintainers. One promising approach to tackle the elevated hunger and reduced energy expenditure that occur with weight loss is the establishment of a high energy flux state in which a high throughput of calories occurs, owing to high daily energy expenditure and matching energy intake. A high flux state compared to a low flux, sedentary state has been found in both observational and experimental studies to result in higher RMR and TEF. Also, energy intake may be more accurately regulated to match energy expenditure in a high flux state. More research is required to determine the magnitude of PAEE required to achieve an effective high energy flux state, whether the intensity of exercise in a high flux state affects the energy gap, and what might be the most advantageous dietary macronutrient composition for a high flux state to narrow the energy gap.
